# Inhibition of Prostaglandin F_2__α_ Receptors Exaggerates HCl-Induced Lung Inflammation in Mice

**DOI:** 10.3390/ijms222312843

**Published:** 2021-11-27

**Authors:** Toko Maehara, Ko Fujimori

**Affiliations:** Department of Pathobiochemistry, Faculty of Pharmacy, Osaka Medical and Pharmaceutical University, 4-20-1 Nasahara, Takatsuki 569-1094, Osaka, Japan; toko.maehara@ompu.ac.jp

**Keywords:** prostaglandin F_2α_ (PGF_2α_), acute lung injury (ALI), lung edema

## Abstract

Acute lung injury (ALI) and acute respiratory distress syndrome (ARDS) are severe respiratory disorders that are caused by aspiration, sepsis, trauma, and pneumonia. A clinical feature of ALI/ARDS is the acute onset of severe hypoxemia, and the mortality rate, which is estimated at 38–50%, remains high. Although prostaglandins (PGs) are detected in the bronchoalveolar lavage fluid of patients with ALI/ARDS, the role of PGF_2α_ in ALI remains unclear. We aimed to clarify the role of PGF_2α_/PGF_2α_ receptor (FP) signaling in acid-induced ALI using an FP receptor antagonist, AL8810. Intratracheal injection of hydrochloric acid (HCl) increased neutrophil migration into the lungs, leading to respiratory dysfunction. Pre-administration of AL8810 further increased these features. Moreover, pre-treatment with AL8810 enhanced the HCl-induced expression of pro-inflammatory cytokines and neutrophil migratory factors in the lungs. Administration of HCl decreased the gene expression of lung surfactant proteins, which was further reduced by co-administration of AL8810. Administration of AL8810 also increased lung edema and reduced mRNA expression of epithelial sodium channel in the lungs, indicating that AL8810 reduced fluid clearance. Furthermore, AL8810 also increased lipopolysaccharide-induced expression of adhesion molecules such as intracellular adhesion molecule-1 and E-selectin in human umbilical vein endothelial cells. These results indicate that inhibition of FP receptors by AL8810 exacerbated HCl-induced ALI.

## 1. Introduction

Acute pulmonary disease, coronavirus disease 2019 (COVID-19), has rapidly evolved into a global pandemic [1]. The number of patients with COVID-19 is over 100 million, and 5 million people have died in just 3 years. Research on and development of therapeutic agents and vaccines against COVID-19 are progressing [2]. However, further research regarding the pathogenesis of COVID-19 and the development of treatments is indispensable. Acute lung injury (ALI) and acute respiratory distress syndrome (ARDS) are severe respiratory diseases caused by aspiration, sepsis, trauma, and pneumonia [3,4]. The clinical characteristics of ALI/ARDS include severe hypoxemia. Morbidity and mortality rates remain high, with an estimated mortality rate of 38–50% [5]. Several clinical studies have shown that pharmacological trials have not been effective in the treatment of ALI/ARDS [6,7]; thus, the development of novel therapeutic strategies for ALI/ARDS is highly necessary. The pathological features of ALI/ARDS include increased neutrophil migration into the alveolar space, alveolar epithelial injury, vascular endothelial injury, and lung edema. Acute inflammation disrupts the endothelial and epithelial barriers [8]. Disruption of the endothelial barriers increases vascular permeability, which results in the efflux of protein-rich fluid into the bronchoalveolar lavage fluid (BALF) [9]. It also reduces alveolar fluid clearance and the production and function of surfactants. Endothelial cell injury upregulates the expression of intracellular adhesion molecule-1 (ICAM-1), which regulates neutrophil adhesion and migration [10]. Infiltrating neutrophils produce reactive oxygen species (ROS) and myeloperoxidase, leading to tissue damage [11].

Prostaglandins (PGs) are lipid mediators that are synthesized from arachidonic acid by the action of cyclooxygenases (COXs) and specific PG synthases [12]. Four major PGs: PGD_2_, PGE_2_, PGF_2α_, and prostacyclin (PGI_2_), exert various biological functions through their specific G protein-coupled receptors [13]. Several reports argue that PGs are involved in the regulation of ALI [14,15,16]. Genetic depletion or pharmacological inhibition of COX-2 suppresses the production of anti-inflammatory mediators and exacerbates acid-induced ALI in mice [14]. Gene deficiency of hematopoietic PGD synthase, a PGD_2_ synthase, enhances neutrophil migration and lung edema in lipopolysaccharide (LPS)-induced ALI in mice [15]. PGE_2_ inhibits fibroblast proliferation and has anti-inflammatory effects in ALI [16]. Thus, these reports show that PGs are involved in the regulation of ALI.

PGF_2α_ is synthesized by PGF synthase, such as aldo-keto reductase (AKR) 1B3, AKR1B7, AKR1B8 [17], and prostamide/PGF synthase. PGF_2α_ exerts various functions including lung inflammation and neutrophil migration through PGF_2α_ receptors (FP). Exogenous administration of PGF_2α_ upregulates the expression of C-X-C motif chemokine ligand 1 (CXCL-1), which is a chemoattractant for neutrophils and promotes neutrophil chemotaxis in endometrial adenocarcinoma [18]. Gene deficiency of FP receptors attenuates bleomycin-induced pulmonary fibrosis in mice [19]. However, the role of PGF_2α_ in ALI remains unclear. In this study, we investigated the role of PGF_2α_/FP receptor signaling in the regulation of HCl-induced ALI in mice.

## 2. Results

### 2.1. Inhibition of FP Receptors Exaggerated HCl-Induced ALI

**Figure 1:**
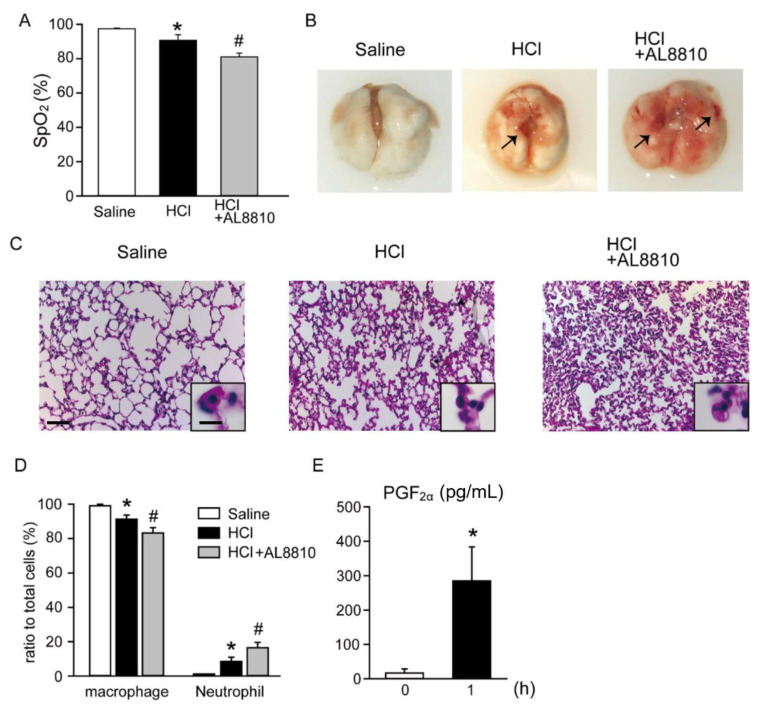
Pretreatment with AL8810 promoted HCl-induced lung inflammation. AL8810 (10 mg/kg, i.p.) was administered to mice 1 h before HCl (0.1 M, 2 µL/g, i.t.) administration. (**A**) SpO_2_ level was used as a measure of pulmonary function (*n* = 3–4). * *p* < 0.05 vs. saline, # *p* < 0.05 vs. HCl. (**B**) Representative images of the lung tissues of saline-, HCl-, and HCl + AL8810-administered mice (*n* = 4–8). (**C**) Representative images of HE-stained sections of the lung tissues of saline-, HCl-, and HCl + AL8810-treated mice (scale bar 50 µm) (*n* = 6–10). (**D**) The cell numbers in BALF were measured at 6 h after administration of saline or HCl (*n* = 5–6). * *p* < 0.05 vs. saline, # *p* < 0.05 vs. HCl. (**E**) The PGF_2α_ levels in BALF were measured by ELISA (*n* = 6). * *p* < 0.05 vs. 0 h.

We first investigated the effect of AL8810 on HCl-induced ALI. The levels of SpO_2_ were measured as an index of pulmonary function. The SpO_2_ levels in saline-treated mice were approximately 98% (Figure 1A). However, the SpO_2_ levels were decreased with HCl treatment, as compared with those of the saline-treated mice, and were further decreased in AL8810-pretreated HCl-administered mice. Administration of HCl-induced lung hemorrhage (indicated by an arrow in Figure 1B, middle panel). Moreover, administration of HCl + AL8810 caused more severe lung hemorrhage than did the administration of HCl alone (indicated by arrows in Figure 1B, right panel). These morphological changes were not observed in the hematoxylin and eosin (HE)-stained sections of saline-treated mice (Figure 1C, left panel). In addition, macrophages were detected in alveolar spaces, which were characterized by a large cytoplasm and round nucleus (Figure 1C, left panel). However, HCl administration resulted in the accumulation of immune cells, mainly neutrophils that were identified by segmented nuclei (Figure 1C, middle panel). Moreover, pretreatment with AL8810 increased HCl-induced neutrophil migration and disruption of alveolar structures (Figure 1C, right panel). Then, we counted the cell population of macrophages and neutrophils in BALF. In the saline-treated mice, macrophages were a major cell population in BALF (Figure 1D). On the other hand, administration of HCl increased the ratio of neutrophils, which was further enhanced in BALF of AL8810-treated mice (Figure 1D). Administration of AL8810 alone did not affect the SpO_2_ level (Figure S1A) or the ratios of macrophages and neutrophils in BALF (Figure S1B), compared with saline-treated mice. At one hour after HCl administration, the PGF_2α_ level in BALF was increased, compared with naïve mice (0 h; Figure 1E). The PGE_2_ level in BALF also tended to increase by HCl administration, compared with naïve mice (Figure S2). Taken together, these results indicate that inhibition of FP receptors exacerbated HCl-induced ALI.

### 2.2. FP Receptor Antagonist Promoted Gene Expression of Pro-Inflammatory Mediators

**Figure 2:**
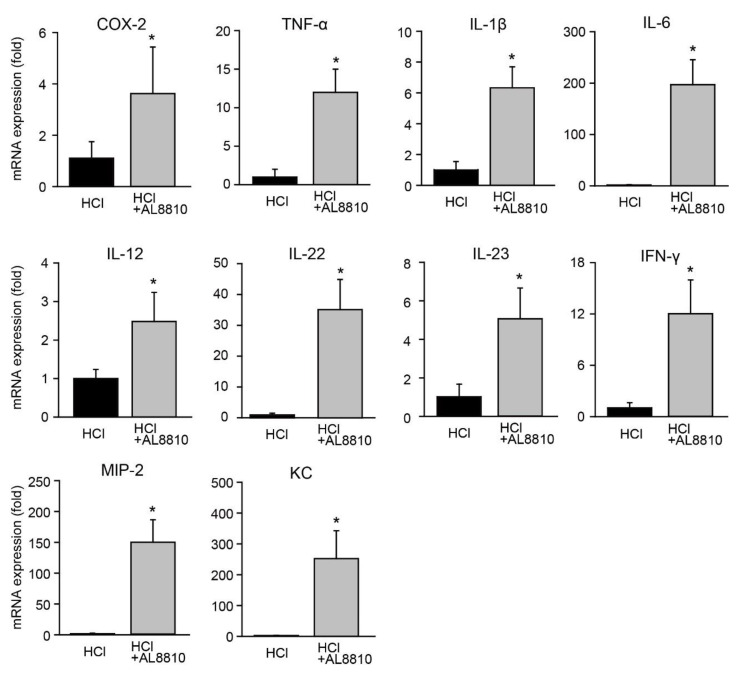
Administration of AL8810 promoted the expression of pro-inflammatory mediators. AL8810 (10 mg/kg, i.p.) was administered to mice at 1 h prior to HCl (0.1 M, 2 µL/g, i.t.) administration. The lung was extracted at 6 h after HCl administration. The gene expression levels of COX-2, TNF-α, IL-1β, IL-6, IL-12, IL-22, IL-23, IFN-γ, MIP-2, and KC in the lungs of HCl- and AL8810-treated mice were measured (*n* = 7). * *p* < 0.05 vs. HCl.

Next, we assessed the gene expression levels of pro-inflammatory mediators associated with ALI. The expression of COX-2 was increased in the lungs of AL8810-pretreated mice, as compared with that in the HCl-administered mice (Figure 2). The gene expression of pro-inflammatory mediators, such as tumor necrosis factor (TNF)-α, interleukin (IL)-1β, and IL-6, was also increased in the lungs of HCl+AL8810-administered mice, as compared with that in HCl-administered mice (Figure 2). IL-12, IL-22, and IL-23 are key regulators of lung inflammation. IL-23 is also known to induce IL-22 production [20]. Bronchial epithelial cells express IL-22, which is associated with the repair of damaged tissues [21]. Pre-treatment with AL8810 elevated the gene expression of IL-12, IL-22, IL-23, and interferon (IFN)-γ in the lungs of mice, as compared with HCl treatment (Figure 2). Keratinocyte-derived chemokine (KC) and macrophage inflammatory protein (MIP)-2 are known as neutrophil chemoattractant chemokines. Pre-administration with AL8810 significantly enhanced these genes’ expression, as compared with HCl treatment (Figure 2). Administration of AL8810 alone did not change the expression levels of TNF-α, IL-1β, and IL-6 (Figure S3). These results suggest that inhibition of FP receptors elevated the expression levels of pro-inflammatory mediators in HCl-treated mice.

### 2.3. Bronchial Epithelial Cells and Macrophage-like Cells Expressed FP Receptors

**Figure 3:**
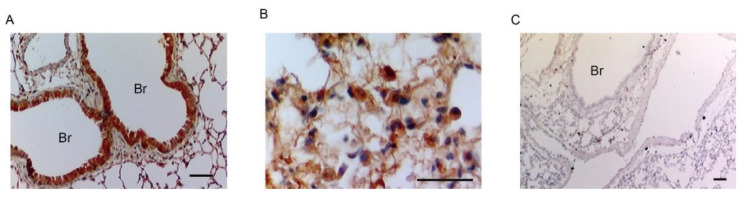
FP receptors were expressed on bronchial epithelium and in alveolar macrophage-like cells. The lung was extracted at 6 h after HCl administration. (**A**,**B**) Representative images of FP receptor-stained lung sections of HCl-treated mice (Br: bronchus, scale bar: 25 µm). (**C**) Representative stained image of the lung without the primary antibody (*n* = 5).

We performed immunostaining analysis to identify the type of cells expressing FP receptors in the lungs of HCl-administered mice. In inflamed lungs, FP receptors were expressed in bronchial epithelial cells (Figure 3A) and alveolar macrophage-like cells, which were characterized by a large cytoplasm and round nucleus (Figure 3B). FP receptors were not detected when no primary antibody was used (Figure 3C).

### 2.4. AL8810 Decreased the Gene Expression of Surfactant Proteins (SFTPs)

**Figure 4:**
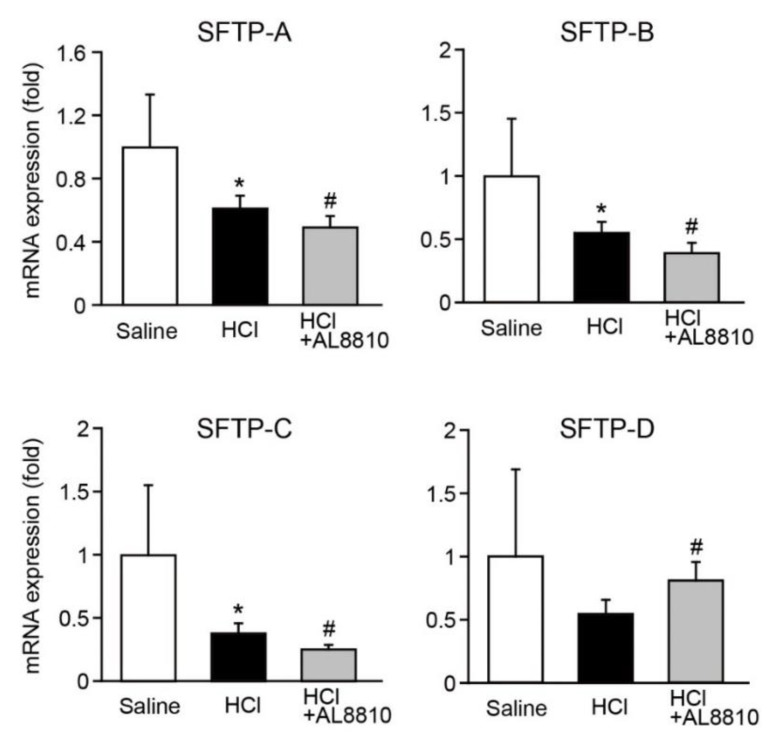
Inhibition of FP receptors decreased the gene expression of SFTP. AL8810 (10 mg/kg, i.p.) was administered to mice at 1 h before HCl (0.1 M, 2 µL/g, i.t.) administration. The lung was extracted at 6 h after administration of saline or HCl. The gene expression levels of SFTP-A, SFTP-B, SFTP-C, and SFTP-D in the lungs of saline-, HCl-, and HCl+AL8810-treated mice were determined by qPCR (*n* = 6–8). * *p* < 0.05 vs. saline, # *p* < 0.05 vs. HCl.

Lung SFTPs are synthesized by epithelial cells to prevent alveolar collapse. SFTPs are key regulators of immune responses in the lungs and alveolar surface tension [22]. Thus, we investigated whether AL8810 affects the expression levels of SFTPs. The expression levels of SFTP-A, SFTP-B, and SFTP-C were significantly decreased in the lungs of HCl-administered mice (Figure 4). Administration of HCl tended to lower the expression level of SFTP-D. Moreover, pre-administration of AL8810 lowered the gene expression of SFTP-A, SFTP-B, and SFTP-C, as compared with that in HCl-administered mice (Figure 4). On the other hand, the expression level of SFTP-D was increased in the lungs of AL8810-treated mice. These results reveal that pharmacological inhibition of FP receptors reduced the gene expression levels of SFTPs in the lungs of HCl-administered mice.

### 2.5. FP Receptor Antagonist Enhanced Lung Edema

**Figure 5:**
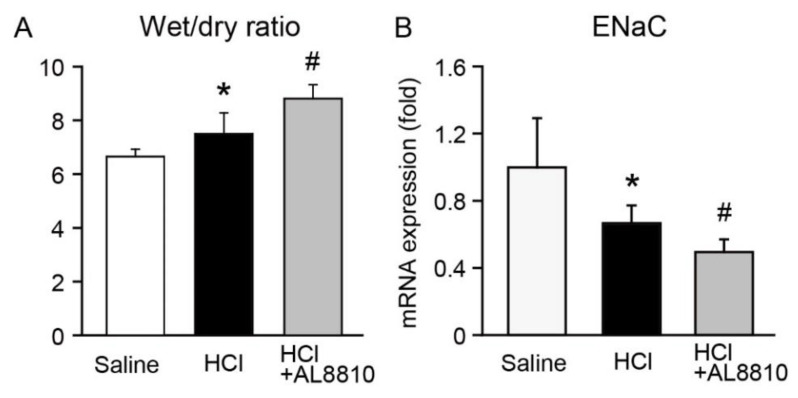
FP receptor antagonist promoted lung edema. AL8810 (10 mg/kg, i.p.) was administered to mice at 1 h before HCl (0.1 M, 2 µL/g, i.t.) administration. (**A**) Lung wet/dry weight ratio was calculated (*n* = 6–7). (**B**) The gene expression levels of ENaC in the lungs of saline-, HCl-, and HCl+AL8810-treated mice were determined by qPCR (*n* = 5–7). * *p* < 0.05 vs. saline, # *p* < 0.05 vs. HCl.

Lung edema is often observed in patients with ALI and is associated with mortality. We thus examined whether an FP receptor antagonist affects lung edema. Administration of HCl increased the wet/dry weight ratio in the lungs, indicating that HCl administration enhanced lung edema (Figure 5A). In addition, pre-administration of AL8810 significantly enhanced HCl-induced lung edema (Figure 5A). The epithelium sodium channel (ENaC) has a pivotal role in lung edema in ARDS/ALI [21]. Activation of ENaC attenuated LPS-induced ALI in mice [23,24]. We examined the effect of AL8810 on the gene expression of ENaC in the lungs. Administration with HCl decreased the gene expression of ENaC, as compared with saline-treated mice, which was further diminished in AL8810-pre-administered mice (Figure 5B).

### 2.6. AL8810 Promoted the Gene Expression of Adhesion Molecules

**Figure 6:**
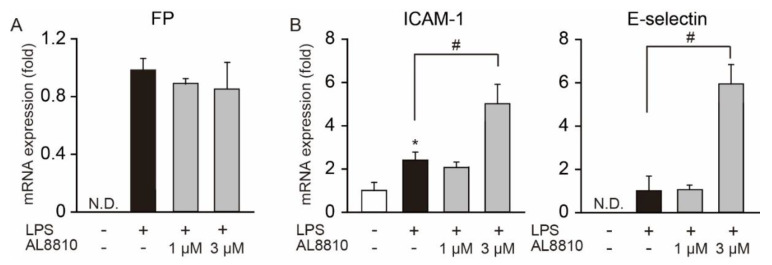
Inhibition of FP receptors on HUVECs enhanced the disruption of endothelium barriers. HUVECs were stimulated with 100 ng/mL LPS for 24 h. AL8810 was treated at 30 min before LPS stimulation (*n* = 3). The expression levels of FP receptors (**A**), ICAM-1, and E-selectin (**B**) were measured by qPCR. * *p* < 0.05 vs. no-treated cells, # *p* < 0.05, as shown by brackets. N.D.: not detected.

In patients with ALI, the expression levels of adhesion molecules in the epithelium, such as ICAM-1 and E-selectin, are increased [25]. An increase in the expression of ICAM-1 and E-selectin causes infiltration of inflammatory cells to tissues from blood vessels [26]. We first examined whether FP receptors are expressed in HUVECs. FP receptors were not expressed in no-treated HUVECs (Figure 6A). LPS stimulation upregulated the mRNA expression of FP receptors, but there were no differences in their expression levels between LPS- and LPS+AL8810-stimulated cells (Figure 6A). We then assessed the effect of AL8810 on the expression of adhesion molecules in HUVECs. Administration of LPS increased the gene expression of ICAM-1 in HUVECs, compared with no-treated cells (Figure 6B). In contrast, E-selectin gene expression was not detected in no-treated cells. Pretreatment with 3 µM AL8810 elevated the expression levels of both ICAM-1 and E-selectin, as compared with those in LPS-stimulated cells. These results suggest that treatment with AL8810 increased the LPS-induced expression levels of ICAM-1 and E-selectin in endothelial cells.

## 3. Discussion

ALI and ARDS are severe respiratory disorders, characterized by severe hypoxemia [4]. According to previous reports, the mortality rate of ALI/ARDS has remained high [5,27]. To investigate the mechanism of ALI/ARDS progression, several animal models, such as mechanical ventilation-, LPS-, hypoxia-, bleomycin-, and acid aspiration-induced ALI, have been used [28]. In addition, it has been reported that there is a gender difference in pneumonia. However, these different animal models show distinct clinical and pathological features and mechanisms of progression of ALI/ARDS. Aspiration of gastric contents is an important risk factor for ALI [29]. Intratracheal injection of acid causes neutrophil migration and injures the airways, alveolar epithelium, and capillary endothelium [28]. Acid aspiration also changes alveolar clearance. In this study, we used an acid aspiration-induced ALI mouse model to elucidate the role of PGF_2α_/FP signaling. Inhibition of FP receptors by AL8810 enhanced HCl-induced pulmonary dysfunction, neutrophil migration, and lung edema, while it decreased SFTP expression. Thus, we showed that PGF_2α_/FP signaling is involved in regulating the progression of acid-induced ALI, which is summarized in Figure S4.

Neutrophils are major immune cells that are observed in BALF and the lungs of patients with ALI/ARDS. In response to pro-inflammatory mediators, neutrophils migrate to the alveolar space [30]. Infiltrating neutrophils cause tissue damage due to the production of histotoxic mediators, such as ROS and neutrophil extracellular traps. There are four steps involved in the recruitment of neutrophils to tissues from blood vessels: (ⅰ) integrin-mediated rolling; (ⅱ) activation; (ⅲ) adhesion to vascular endothelium; and (ⅳ) transendothelial migration [31,32]. Upregulation of adhesion molecules such as ICAM-1 and E-selectin in the vascular endothelium induces neutrophil migration [26]. In an in vitro assay, exogenous PGF_2α_ administration upregulated CXCR1 expression and increased neutrophil migration [18]. However, we showed that the inhibition of FP receptors promoted neutrophil migration into the lungs and BALF (Figure 1C,D). We also demonstrated that AL8810 increased the LPS-induced gene expression of ICAM-1 and E-selectin in HUVECs (Figure 6). Taken together, these results indicate that inhibition of FP receptors in the vascular endothelium increased the expression of molecular adhesion factors, which promoted neutrophil migration into the lungs. Adhesion molecules are also expressed in bronchial epithelium [33]. Upregulation of the expression of adhesion molecules promotes leucocyte infiltration into bronchi. It is possible that inhibition of FP receptors in bronchial epithelium also regulates the expression of adhesion molecules such as ICAM-1 and E-selectin, which increases neutrophil migration into the lungs. Further investigation is needed to clarify the regulatory mechanism of PGF_2α_/FP signaling on neutrophil migration.

Pulmonary surfactant is composed of lipids and proteins, and it is produced in type 2 alveolar epithelial cells [34]. Surfactant lipids are synthesized in the endoplasmic reticulum. The components of alveolar surfactant proteins are SFTP-A, SFTP-B, SFTP-C, and SFTP-D. The lack of surfactant lipids leads to death in patients with ARDS [35]. Intratracheal injection of SFTP-B and SFTP-C significantly improves mortality and morbidity in patients with ARDS [36]. SFTP-A and SFTP-D regulate alveolar surface tension. Gene deficiency of SFTP-B is lethal due to atelectasis and respiratory failure [37]. Inhibition of FP receptors decreased the expression of SFTP-A, SFTP-B, SFTP-C, and SFTP-D (Figure 4). We assessed the mRNA expression of SFTP but not the protein expressions. Previous studies have shown that mRNA expression patterns are not necessarily correlated with protein expression [38,39]. Further studies are needed to clarify the details of the regulatory mechanism of PGF_2α_/FP signaling on the production of pulmonary surfactant, including the control of these proteins’ expression.

FP receptors were expressed in the bronchial epithelium and macrophage-like cells (Figure 3). It has been reported that alveolar macrophage-derived pro-inflammatory mediators are initiators of inflammation in ALI [40]. We previously reported that the inhibition of FP receptors promoted the gene expression of pro-inflammatory mediators such as IL-1β, TNF-α, and inducible nitric oxide synthase in LPS + IFN-γ-stimulated macrophages [41]. In this model, inhibition of FP receptors in alveolar macrophages may produce pro-inflammatory mediators, which results in increased neutrophil migration. The airway epithelium plays a critical role in fluid transport. Patients with ALI/ARDS with protein-rich fluid in the alveolar space show higher mortality rates than those without protein-rich fluid [3]. ENaC and cystic fibrosis transmembrane regulator (CFTR) are major transporters that regulate alveolar fluid clearance [42]. ENaC and CFTR are expressed in the epithelium and endothelium. PGF_2α_ enhances ENaC activity in cortical collecting duct cells [43]. In this HCl-induced ALI model, administration with AL8810 enhanced HCl-induced lung edema (Figure 5A). Administration with AL8810 also decreased the gene expression of ENaC in the lungs (Figure 5B). Therefore, it is possible that the FP receptors expressed in the bronchial epithelium are involved in the regulation of ENaC activation, which maintains fluid clearance.

In the present study, we showed that inhibition of FP receptors increased the gene expression of pro-inflammatory mediators, neutrophil migration into BALF, and lung edema in HCl-administered mice. This is the first report that PGF_2α_/FP signaling is important in regulating the progression of HCl-induced ALI.

## 4. Conclusions

Inhibition of FP receptors increased the gene expression of pro-inflammatory mediators and neutrophil migration into the lung, and it enhanced lung permeability and disruption of vascular endothelial cells in HCl-administered mice. Moreover, pharmacological inhibition of FP receptors elevated the expression of adhesion molecules in HUVECs. Furthermore, inhibition of FP receptors promoted the endothelial barrier dysfunction and the expression of adhesion molecules, which exacerbated HCl-induced ALI in mice. Therefore, PGF_2α_/FP signaling is important in regulating the progression of HCl-induced ALI. In addition, FP receptor agonists may have future pharmaceutical potential to treat ALI in human patients.

## 5. Materials and Methods

### 5.1. Mouse Model

All experiments and animal care were conducted in accordance with the institutional guidelines of the Osaka Medical and Pharmaceutical University. C57BL/6J mice (8-week-old females; Japan SLC, Shizuoka, Japan) were used to induce lung inflammation. Mice were anesthetized with 3% (*v*/*v*) isoflurane (FUJIFILM Wako Pure Chemical, Osaka, Japan) and 50 mg/kg pentobarbital sodium (Nacalai Tesque, Kyoto, Japan). Acute lung injury was induced by intratracheal (i.t.) administration of HCl (0.1 M, 2 µL/g). AL8810 (10 mg/kg in 1% DMSO, Cayman Chemical, Ann Arbor, MI, USA) was administered intraperitoneally at 1 h before HCl administration. Mice were randomly divided into three groups: administration of saline (1% DMSO in 200 µL saline, i.p.), HCl (1% DMSO in 200 µL saline, 30 min before HCl administration, i.p.), and HCl+AL8810 (*n* = 3–5 per group/experiment, repeated 4 times).

### 5.2. Measurement of Saturation of Peripheral Oxygen (SpO_2_)

At six hours after saline or HCl administration, SpO_2_ levels were measured using a mouseOx pulse oximeter (Starr Life Sciences, Oakmont, PA, USA). The pulse oximeter was placed around the neck of mice that were anesthetized using isoflurane (induction; 3% (*v*/*v*), maintenance; 1.5–2% (*v*/*v*)). The levels of SpO_2_ were measured every second for 1 min while maintaining the breathing rate at 120–160 breaths per minute (bpm). The data are represented as the average of the SpO_2_ levels measured for 1 min.

### 5.3. RNA Extraction and Quantitative PCR (qPCR)

At six hours after saline or HCl administration, total RNA was extracted using RNAiso Plus (Takara Bio, Kyoto, Japan), and the concentration of total RNA was adjusted to 0.5 μg/µL. RNA was reverse-transcribed to cDNA using a random 9-mer primer (Takara Bio) and ReverTra Ace (Toyobo, Osaka, Japan). Reverse transcription was carried out at 30 °C for 10 min, followed by incubation at 42 °C for 60 min. Subsequently, the reaction was stopped by heat inactivation at 95 °C for 5 min. The cDNAs were used for quantitative analysis of target gene expression levels using a Power SYBR Green PCR Master Mix (Thermo Fisher Scientific, Waltham, MA, USA) and a LightCycler 96 System (Roche Life Science, Penzberg, Germany). The mRNA expression levels were normalized to 18S rRNA levels and calculated using the ΔΔCt method. Details of the primer sets used in the qPCR analysis are shown in Table 1.

### 5.4. Calculation of Lung Wet/Dry Weight Ratio

At six hours after administration of saline or HCl, the lung was extracted and weighed (wet weight). Then, the lung was dried at 50 °C, and after 24 h, the dried weight was measured (dry weight). The lung wet/dry weight ratio was used as an index of lung water accumulation.

### 5.5. Cell Counting and Cell Differentiation in BALF

Cells in BALF were attached to glass slides by cytospin centrifugation at 2000 rpm for 5 min (Thermo Fisher Scientific). The glass slides were stained with Diff Quick (Sysmex Corporation, Hyogo, Japan) according to the manufacturer’s instructions. The cell populations were counted for 4 randomly selected fields.

### 5.6. Measurement of PGF_2α_ and PGE_2_ Levels

PGF_2α_ and PGE_2_ levels in BALF were measured using the respective ELISA kits (Cayman Chemical) according to the manufacturer’s instructions. Briefly, BALF was centrifuged at 450× *g* for 3 min at 4 °C, and the resulting supernatant was used for the measurement of PGF_2α_ and PGE_2_ levels.

### 5.7. Immunostaining

At six hours after saline or HCl administration, the lung was extracted. The lung was fixed in 4% (*w*/*v*) paraformaldehyde for 48 h and embedded in paraffin. The tissue block was cut into 4 µm sections. To perform the antigen retrieval, the glass slides were incubated with sodium citrate (10 mM, pH 6.0) for 10 min at 95 °C, followed by incubation for 30 min at 25 °C. To block endogenous peroxidase activity, the glass slides were incubated with 3% (*v*/*v*) hydrogen peroxidase for 10 min at 25 °C. After washing, to block the non-specific protein adherence to sections and permeabilize the lung, the sections were incubated with 10% (*v*/*v*) normal goat serum and 0.1% (*v*/*v*) Triton-X in PBS for 15 min at 25 °C. Then, the glass slides were washed with PBS and incubated with anti-FP receptor antibody (Cayman Chemical) [44] for 16 h at 4 °C. After washing with PBS, the glass slides were incubated with biotin-conjugated anti-rabbit IgG secondary antibody (Santa Cruz Biotechnology, Dallas, TX, USA) for 3 h at 25 °C. The glass slides were incubated with avidin-biotin complex reagent (Nacalai Tesque) for 30 min at 25 °C, followed by staining using a DAB substrate staining kit (Nacalai Tesque). Nuclear counterstaining was performed with hematoxylin as described previously [26].

### 5.8. Cell Culture

Human umbilical vein endothelial cells (HUVECs) were obtained from the JCRB cell bank (National Institutes of Biomedical Innovation, Health and Nutrition, Osaka, Japan). HUVECs were cultured in Endothelial Cell Basal Medium (EBM)-2 medium with Endothelial Cell Growth Medium 2, containing 2% (*v*/*v*) fetal bovine serum (PromoCell GmbH, Heidelberg, Germany), 100 U/mL penicillin, and 100 μg/mL streptomycin (Nacalai Tesque) at 37 °C in an atmosphere of 5% CO_2_. The HUVECs were stimulated with 100 ng/mL LPS (Sigma, St. Louis, MO, USA) for 24 h. AL8810 (1 and 3 µM) was treated at 30 min before LPS stimulation.

### 5.9. Statistical Analysis

The data are presented as mean ± S.D. Statistical analysis between two groups was conducted using Student’s *t*-test. For more than three groups, statistical analysis was conducted using a one-way ANOVA. Statistical significance was set at *p* < 0.05.

## Figures and Tables

**Table 1:** Nucleotide sequences of primers used in real-time PCR analysis.

Gene	Forward	Reverse	Product Length (bp)
COX-2	GATGCTCTTCCGAGCTGTG	GGATTGGAACAGCAAGGATTT	75
IFN-γ	GCGTCATTGAATCACACCTG	TGAGCTCATTGAATGCTTGG	129
IL-1β	AGTTGACGGACCCCAAAAG	AGCTGGATGCTCTCATCAGG	75
IL-6	GCTACCAAACTGGATATAATCAGGA	CCAGGTAGCTATGGTACTCCAGAA	78
IL-12	AGCAGTAGCAGTTCCCCTGA	AGTCCCTTTGGTCCAGTGTG	88
IL-22	GTCAACCGCACCTTTATGCT	GAACAGTTTCTCCCCGATGA	84
IL-23	AATAATGTGCCCCGTATCCA	AGGCTCCCCTTTGAAGATGT	144
KC	CCGAAGTCATAGCCACACTCAA	GCAGTCTGTCTTCTTTCTCCGTTAC	128
MIP-2	AGACAGAAGTCATAGCCACTCTCAAG	CCTCCTTTCCAGGTCAGTTAGC	126
ENaC	TGTGCATTCACTCCTGCTTC	ACCCTTGGGCTTAGGGTAGA	80
SFTP-A	AAGGGAGAGCCTGGAGAAAG	AGGACTCCCATTGTTTGCAG	113
SFTP-B	TTGTCCTCCGATGTTCCACT	GGCATAGCCTGTTCACTGGT	151
SFTP-C	CAGCTCCAGGAACCTACTGC	TCGGACTCGGAACCAGTATC	160
SFTP-D	AAGCTGCATTGTTCCCTGAT	GCTGTATGGCAGCATTCTCA	156
TNF-α	TGCCTATGTCTCAGCCTCTTC	GAGGCCATTTGGGAACTTCT	117
ICAM-1	CCTTCCTCACCGTGTACTGG	AGCGTAGGGTAAGGTTCTTGC	90
E-selectin	ACCAGCCCAGGTTGAATG	GGTTGGACAAGGCTGTGC	89
18S rRNA	GGGAGCCTGAGAAACGGC	GGGTCGGGAGTGGGTAATTT	68

## Data Availability

The data presented in this study are available on request from the corresponding author.

## Supplementary Materials

Supplementary materials can be found at https://www.mdpi.com/article/10.3390/ijms222312843/s1.
