# Supplementary material for: Inhibition of Prostaglandin F2α Receptors Exaggerates HCl-Induced Lung Inflammation in Mice
Source: Int J Mol Sci. 2021 Nov 27;22(23):12843. doi: 10.3390/ijms222312843 (PMC8657597; doi:10.3390/ijms222312843)
Supplement: Supplementary file 1 [file ijms-22-12843-s001.zip › ijms-1459053-supplementary.pdf]

## SUPPLEMENTARY MATERIALS

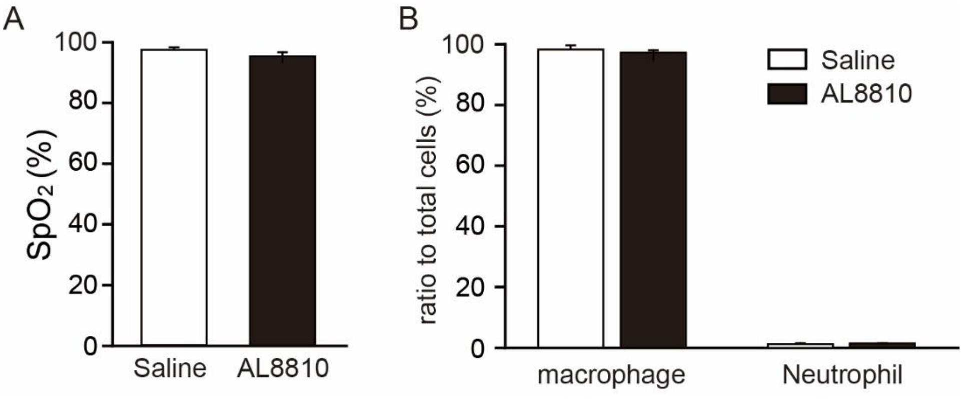


**Fig. S1. AL8810 alone did not affect $SpO_2$ level and cell population in BALF.**

AL8810 (10 mg/kg, i.p.) was administered to mice. (A) At six h after saline or AL8810 administration, $SpO_2$ level was measured as a pulmonary function (n=4). (B) The cell number in BALF were measured at 6 h after administration of saline or AL8810 (n=4).

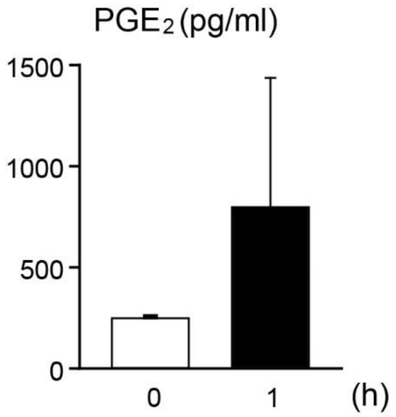


**Fig. S2. HCl administration promoted $PGE_2$ production in BALF.**

The $PGE_2$ levels in BALF were measured by ELISA (n=6).

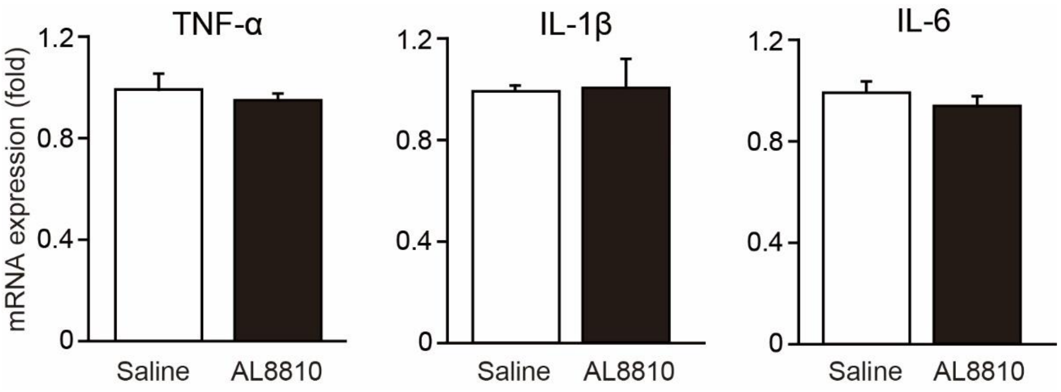


**Fig. S3. Administration of AL8810 did not affect the expression of pro-inflammatory mediators.**
AL8810 (10 mg/kg, i.p.) was administered to mice. At six h after saline or AL8810 administration, lung was extracted. The gene expression levels of TNF-α, IL-1β, and IL-6 in the lungs of saline- or AL8810-treated mice were measured (n=4).

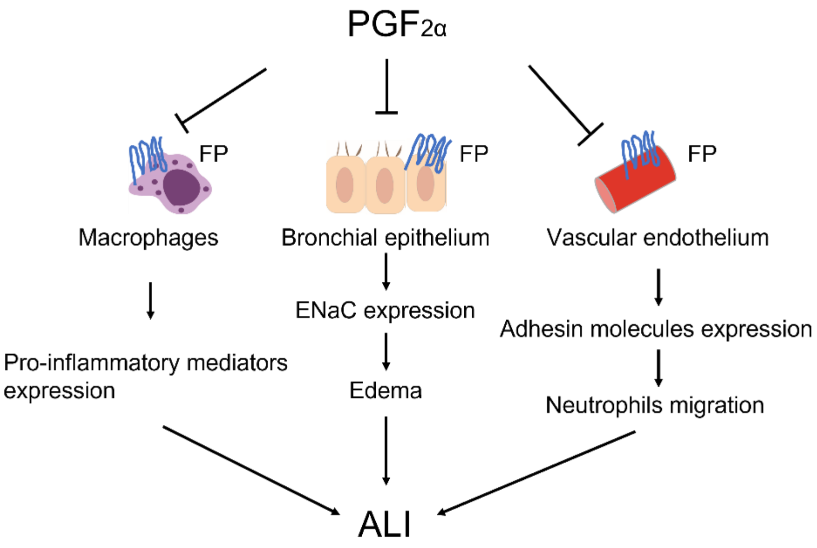


**Fig. S4. Proposed scheme of role of FP receptors in ALI.**
Inhibition of FP receptors enhanced HCl-induced ALI. FP receptors were expressed in macrophage-like cells, bronchial epithelium, and vascular endothelium. Inhibition of FP receptors in macrophages increased the expression of pro-inflammatory mediators. Administration with HCl decreased the gene expression of ENaC, as compared with saline-treated mice, which was further diminished in AL8810-pre-administered mice. Thus, FP receptors expressed in bronchial epithelium is involved in the regulation of the expression of ENaC. Inhibition of FP receptors expressed in vascular endothelial cells upregulated the expression of adhesion molecules and induced leukocytes infiltration.
